# Relevance of treatment‐free remission recommendations in chronic phase chronic leukemia patients treated with frontline tyrosine kinase inhibitors

**DOI:** 10.1002/cam4.3921

**Published:** 2021-05-14

**Authors:** Gabriel Etienne, Carole Faberes, Fréderic Bauduer, Didier Adiko, François Lifermann, Corinne Dagada, Caroline Lenoir, Anna Schmitt, Emilie Klein, Marie‐Pierre Fort, Fontanet Bijou, Beatrice Turcq, Fanny Robbesyn, Françoise Durrieu, Laura Versmée, Samia Madene, Marius Moldovan, Sandrine Katsahian, Anais Charles‐Nelson, Axelle Lascaux, François‐Xavier Mahon, Stéphanie Dulucq

**Affiliations:** ^1^ Département d’Hématologie Institut Bergonié Bordeaux France; ^2^ Institut National de la Santé et de la Recherche Médicale U1218 ACTION Université de Bordeaux Bordeaux France; ^3^ Groupe France Intergroupe des Leucémies Myéloïdes Chroniques Hôpital Haut‐Lévêque Pessac France; ^4^ Service d’Hématologie Centre Hospitalier Côte Basque Bayonne France; ^5^ Collège des Sciences de la Santé Université de Bordeaux Bordeaux France; ^6^ Service d’Hématologie Centre Hospitalier de Libourne Libourne France; ^7^ Service de Médecine Interne Centre Hospitalier de Dax‐Côte d’Argent Dax France; ^8^ Service d’Oncologie‐Hématologie Centre Hospitalier de Pau Pau France; ^9^ Service d’Hémato‐Oncologie Radiothérapie Polyclinique Bordeaux Nord Aquitaine Bordeaux France; ^10^ Laboratoire d’Hématologie Hôpital Haut Lévêque Centre Hospitalier Universitaire de Bordeaux Pessac France; ^11^ Centre National de la Recherche Scientifiue SNC 5010 Bordeaux France; ^12^ Service de Médecine Interne et Hématologie Centre Hospitalier Intercommunal Mont‐de‐Marsan – Pays des Sources Mont de Marsan France; ^13^ Hôpital de jour Hématologie‐Oncologie Centre Hospitalier de Périgueux Périgueux France; ^14^ Unité de Recherche Clinique et Centre Investigation Clinique‐Epidémiologie Hôpitaux Universitaires Paris‐Ouest Hôpital Européen Georges Pompidou Assistance Publique‐Hôpitaux de Paris Paris France; ^15^ Université Paris 5 Institut National de la Santé et de la Recherche Médicale Centre de Recherche des Cordeliers Equipe 22 Paris France; ^16^ INSERM Centre d'Investigation Clinique 1418 Module Epidémiologie Clinique Paris France; ^17^ Service Des Maladies du Sang Hôpital Haut Lévêque Centre Hospitalier Universitaire de Bordeaux Pessac France

**Keywords:** molecular recurrence‐free survival, recommendations, tyrosine kinase inhibitor discontinuation

## Abstract

**Background:**

Tyrosine kinase inhibitors (TKI) can be safely discontinued in chronic phase chronic myeloid leukemia (CP‐CML) patients who had achieved a sustained deep molecular response. Based on the results of discontinuation trials, recommendations regarding patient selection for a treatment‐free remission (TFR) attempt had been proposed. The aims of this study were to evaluate the rate of patients eligible for TKI discontinuation and molecular recurrence‐free survival (MRFS) after stop according to recommendations.

**Methods:**

Over a 10‐year period, newly diagnosed CP‐CML patients and treated with first‐line TKI in the nine French participating centers were included. Eligibility to treatment discontinuation and MRFS were analyzed and compared according to selection criteria defined by recommendations and first‐line treatments.

**Results:**

From January 2006 to December 2015, 398 patients were considered. Among them, 73% and 27% of patients received imatinib or either second or third generation tyrosine kinase inhibitors as frontline treatment, respectively. Considering the selection criteria defined by recommendations, up to 55% of the patients were selected as optimal candidates for treatment discontinuation. Overall 95/398 (24%) discontinued treatment. MRFS was 51.8% [95% CI 41.41–62.19] at 2 years and 43.8% [31.45–56.15] at 5 years. Patients receiving frontline second‐generation TKI and fulfilling the eligibility criteria suggested by recommendations had the lowest probability of molecular relapse after TKI stop when compare to others.

**Conclusion:**

One third of CP‐CML patients treated with TKI frontline fulfilled the selection criteria suggested by European LeukemiaNet TFR recommendations. Meeting selection criteria and second‐generation TKI frontline were associated with the highest MRFS.

## Graphical abstract

Tyrosine kinase inhibitors (TKI) can be safely discontinued in chronic phase chronic myeloid leukemia patients who had achieved a sustained deep molecular response. Several recommendations regarding the optimal selection of the patients before stop have been proposed. Based on these recommendations, we estimate that one third of the patients treated with frontline TKI will meet these criteria. Moreover, patients treated with frontline second generation TKI and selected as optimal candidate according to these recommendations have the highest probability of molecular recurrence‐free survival when compared to others.

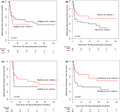

## 1 INTRODUCTION

Following the results of the first two large studies evaluating the possibility of imatinib (IMA) discontinuation in chronic phase chronic myeloid leukemia (CP‐CML) patients who had achieved a sustained deep molecular response (DMR), numerous experiences of tyrosine kinase inhibitor (TKI) cessation are now available.^1^, ^2^, ^3^ Currently, thousands CML patients have discontinued TKI. Approximately half of them have prolonged treatment‐free remission (TFR). Several predictive factors have been correlated to the risk of molecular recurrence (MolRec) after TKI stop. Among these, depth and duration of a molecular response and TKI duration before TKI cessation are the most relevant. However, rather than an accurate prediction of MolRec after TKI stop, these criteria ensure that TKI resumption will lead to a second DMR in most if not all the patients. Given the recent report of infrequent progression events after TKI discontinuation, selecting patients who will be optimal candidates for TFR experience outside a clinical trial and in daily clinical practice remains a significant challenge.^4^, ^5^ Over the past 4 years several recommendations regarding the selection of the patients for a TFR program have been proposed.^6^, ^7^, ^8^, ^9^ While CP of the disease at diagnosis and during follow‐up and typical *BCR*‐*ABL1* transcript are conditions shared by all of these recommendations, TKI type and duration, depth and duration of DMR and CML history in terms of non‐optimal response or switch unrelated to TKI intolerance, support some slight differences. Whether these differences have an impact on TFR eligibility and TFR rate remains to be dertermined. The aims of this retrospective and observational study were to evaluate the proportion of eligible patients to TFR and to estimate the molecular recurrence‐free survival (MRFS) after TKI cessation in CP‐CML according to the patient selection criteria suggested in these recommendations.

## 2 METHODS

### 2.1 Patients

Over a 10‐year period (January 2006 to December 2015), patients of 18 year‐old or more, diagnosed in CP‐CML and treated with TKI at any initial daily dose in the nine participating centers in southwest France were included in this study. Patients were identified in the southwest CML register approved by the Comission Nationale Informatique et Libertés (CNIL, Paris, France; CNIL approval DR‐2015–384). All living patients have been informed and have not expressed their opposition to be included in this retrospective and observational study according to the current French regulations. The study has been approved by the Institut Bergonié institutional review board.

### 2.2 Definitions

Phase of the disease, Sokal and EUTOS long‐term survival (ELTS) risk scores, responses to TKIs as first‐line treatment were defined according to the European LeukemiaNet (ELN) recommendations.^9^, ^10^ Molecular response was assessed by reverse transcription quantitative polymerase chain reaction (RT‐qPCR) and reported as ratios of *BCR*‐*ABL1* to *ABL1* standardized to the international scale (IS) (*BCR*‐*ABL^IS^*) according to previously reported recommendations.^11^, ^12^ *BCR*‐*ABL1*/*ABL1* assessments were performed every 3–4 months and MR4 and MR4.5 were defined as 4‐log reduction or *BCR*‐*ABL^IS^* ≤0.01% and 4.5‐log reduction or *BCR*‐*ABL^IS^* ≤0.0032%, respectively. Each molecular response level has to be confirmed on two consecutive analyses at least two months apart. Date of each level corresponds to the first date of occurrence. Molecular responses were defined as sustained if no *BCR*‐*ABL^IS^* demonstrated a MR4 loss during the observational period for MR4, and for MR4.5 if no more than one *BCR*‐*ABL^IS^* assessment on a twelve‐month period demonstrated a MR4.5 loss with retained MR4.

Molecular follow‐up after TKI cessation was performed as recommended.^6^, ^7^, ^8^, ^9^ MolRec after TKI discontinuation was defined either as a confirmed positivity of *BCR*‐*ABL^IS^* with an increase of 1‐log on two consecutive assessments or MMR loss. Considering the selection criteria proposed by Hughes et al.,^6^ and Hochhaus et al.,^9^ the "green" criteria and the "optimal" situation in patients fulfilling the mandatory requirements for TKI discontinuation were retained for the analyses, respectively.^6^, ^9^ Selection criteria according to the proposed recommendations are summarized in Table 1.

**TABLE 1:** Proposed selection criteria for optimal tyrosine kinase discontinuation according to Hughes et al.,^6^ Rea et al.,^7^ Radich et al.,^8^ and Hochhaus et al.^9^

Reference	Hughes et al. Blood 2016	Rea et al. Cancer 2018	Radich et al. J Natl Compr Canc Netw. 2018	Hochhaus et al. Leukemia 2020
Proposed selection criteria for optimal TKI discontinuation	CP only	CP only	CP only	CP‐CML in first CP
	*BCR*‐*ABL1* transcript e13a2 or e14a2	*BCR*‐*ABL1* transcript e13a2 or e14a2	Prior evidence of quantifiable *BCR*‐*ABL1* transcript	Typical *BCR*‐*ABL1* transcript (e13a2, e14a2)
	MR4.5 sustained >24 months	MR4.5 sustained >24 months	MR4 sustained ≥24 months	MR4 > 3 years or MR4.5 > 2 years
	TKI duration >8 years	TKI duration >5 years	TKI duration ≥3 years	TKI duration >5 years
	Optimal response to first‐line TKI	No prior AHSCT, progression, resistance, suboptimal response, or warning	No prior history of accelerated or blast phase CML	First‐line TKI or 2nd line if change related to intolerance
	Sokal non high	≥18 years at TKI discontinuation	Age ≥18 years	No prior treatment failure

Abbreviations: AHSCT, allogenic hematopoietic stem cell transplantation; CP, chronic phase; TKI, tyrosine kinase inhibitor.

Eligibility to TFR according to the recommendations was performed as follow. Patients were selected first on the basis of duration and depth of molecular response as required, followed by TKI duration and finally for the selected patient by baseline characteristics (atypical *BCR*‐*ABL1* transcript, Sokal risk score) and CML history (non‐optimal response according to the 2013 ELN recommendations, TKI switch unrelated to intolerance).^10^

### 2.3 Statistical analyses

Patients were classified into two groups for each recommendation based on whether they met the corresponding criteria. Molecular recurrence‐free survival (MRFS) was measured from the date of TKI discontinuation to the date of MolRec or last follow‐up for patients who did not relapse. MRFS with their 95% confidence interval (95% CI) were estimated by Kaplan Meier Method, and between‐group comparisons were performed using the logrank test. Several deaths were observed in DMR patients without MolRec, molecular recurrence‐free remission (MRFR) was assessed by Fine and Gray method, considering deaths as competing risks. The impact of TKI frontline (IMA vs 2‐G TKI) associated with the presence or absence of eligibility criteria on the occurrence of MolRec was secondly analyzed. Cumulative incidences (CIn) of re‐acquisition of MMR, MR4 and MR4.5 in patients who restarted TKI following MolRec, were measured from the date of TKI resumption to the date of achieving a second DMR. CIn of MMR was assessed only in patients who lost MMR before TKI resumption. As some patients died before achieving some second molecular response, CIn were analyzed using Fine and Gray method, considering deaths as competing risks.

All statistical analyses were carried with R software and IBM^®^ SPSS software, version 22 (IBM Corp.). The statistical significance level was (two‐sided) 0.05.

## 3 RESULTS

### 3.1 Patients

From January 2006 to December 2015, 398 CP‐CML patients diagnosed in the nine participating centers in southwest France have been included in this study.

Baseline characteristics of the study population have been previously reported.^13^ Briefly, the median age at diagnosis was 61.7 years (range, 18.6 to 90.2 years), 58% of patients were male, and Sokal and ELTS risk scores were high in 19% and 13% of them, respectively. Three (0.7%) patients harbored an atypical *BCR*‐*ABL1* transcript and 30 (7.5%) patients have an additional chromosome abnormality considered as "major route" in 12 (3%) of them. First‐line TKI was IMA and second or third generation TKI (2‐3G TKI) in 291 (73%) and 107 (27%) patients, respectively. With a median follow‐up of 7 years (range, 0.6 to 13.8 years), 182 (46%) patients had achieved a MR4.5 sustained at least 24 months.

### 3.2 Eligibility to treatment‐free remission according to selection criteria recommendations

**FIGURE 1:**
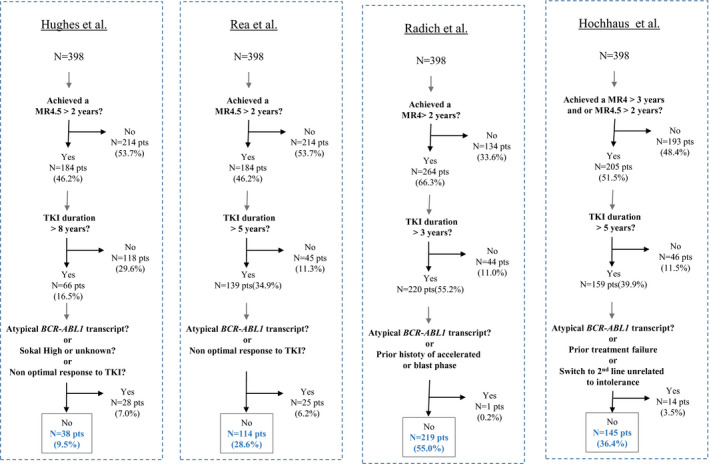
Eligibility to treatment free remission according to selection criteria proposed in Hughes et al.,^6^ Rea et al.,^7^ Radich et al.,^8^ and Hochhaus et al.^9^ in de novo CP‐CML patients (*N* = 398 pts)

Based on the selection criteria suggested by Hughes et al.,^6^ Rea et al.,^7^ Radich et al.,^8^ and Hochhaus et al.,^9^ 38 (9.5%), 114 (28.6%), 219 (55.0%) and 145 (36.4%) patients were optimal candidates to TFR, respectively (Figure 1). All the patients selected as optimal candidates with the criteria proposed by Rea et al.^7^ were all positively selected by Hochhaus et al.^9^ recommendation criteria. Among the 145 patients qualified as optimal according to Hochhaus et al. criteria,^9^ 31 patients were not considered as optimal for TKI cessation according to Rea et al. criteria.^7^ Among them, 15 patients have a non‐optimal response to TKI frontline (*BCR*‐*ABL^IS^* >10% at 3 months and >0.1%‐1% at 12 months, *n* = 4; >0.1%–1% at 12 months, *n* = 11) and 16 patients failed to achieved an MR4.5 sustained for at least two years but maintained an MR4 sustained at least three years, despite TKI duration longer than five years for all them.

### 3.3 Molecular recurrence‐free survival

Among the 398 patients, 95 (23.8%) patients discontinued TKI with a TFR attempt. Baseline and ongoing characteristics of the study population is presented in Table 2. Among them 10/95 patients died of CML‐unrelated causes (secondary neoplasm, *n* = 2; cardiovascular diseases, *n* = 2; non‐cardiac chronic organ failures, *n* = 5; infectious disease, *n* = 1). The median age at death was 83.1 years (range: 66.0–92.4). Four patients died without MolRec at 8.0, 8.7, 10.3 and 54.5 months after TKI stop. The remaining 6 patients died after MolRec with a median time from TKI stop and TKI resumption following MolRec to the date of death of 45.3 months (range: 9.9–73.0) and 32.0 months (range: 3.4–60.3), respectively. With a median follow‐up of 36.46 months (range: 4.16–121.93) after TKI discontinuation, 44/95 (46.32%) patients had molecular recurrence consisting in MMR loss (*n* = 38) or confirmed loss of MR4 with an increase of *BCR*‐*ABL^IS^* of at least one log (*n* = 6) increase. Then MRFS was 51.8% [95% CI: 41.41–62.19] at 2 years and 43.8% [95% CI: 31.45–56.15] at 5 years. The median follow‐up for patients without MolRec was 50.6** **months (ranged: 4.2–121.9). No resumption of treatment in DMR was observed and no CML event progression was reported.

**TABLE 2:** Baseline and ongoing characteristics of the study population (*N* = 95 pts)

Variables	*N* = 95
Female gender, *n* pts, (%)	53 (55.8)
Median age at diagnosis, years (range)	62.6 (19.2–85.6)
Sokal Risk Score, *n* pts (%)	
Low	23 (24.2)
Intermediate	47 (49.5)
High	21 (22.1)
Unknown	4 (4.2)
ELTS Risk Score, *n* pts (%)	
Low	53 (55.8)
Intermediate	23 (24.2)
High	13 (13.7)
Unknown	6 (6.3)
BCR‐ABL transcript type, *n* pts (%)	
B2A2	31 (32.6)
B3A2	54 (56.8)
B2A2+B3A2	3 (3.2)
M	7 (7.4)
ACA at diagnosis (major), *n* pts	5 (5.3)
IMA first‐line, *n* pts (%)	71 (74.7)
2‐G TKI first‐line, *n* pts (%)	24 (25.3)
Achieved MR4.5, *n* pts (%)	87 (91.6)
Median time from TKI start to MR4.5, months (range)	22 (2.6–88.0)
Achieved MR4.5 sustained at least 24 months, *n* pts	85 (89.5)
Median MR4.5 duration, months (range)	44 (13.1–113.4)
TKI First‐line at TKI discontinuation, *n* pts (%)	75 (78.9)
Median Time from TKI start to TKI discontinuation, months (range)	72.6 (33.6–134.4)
Median follow‐up after TKI stop, months (range)	36.5 (4.2–121.9)

Abbreviations: 2G TKI, second‐generation TKI; ACA, additional cytogenetic abnormalities; ELTS, EUTOS Long‐Term Survival; IMA, imatinib; MR4, Molecular Response 4 log; MR4.5, Molecular Response 4.5 log; TKI, tyrosine kinase inhibitor.

### 3.4 Molecular recurrence‐free survival according to selection criteria recommendations

**FIGURE 2:**
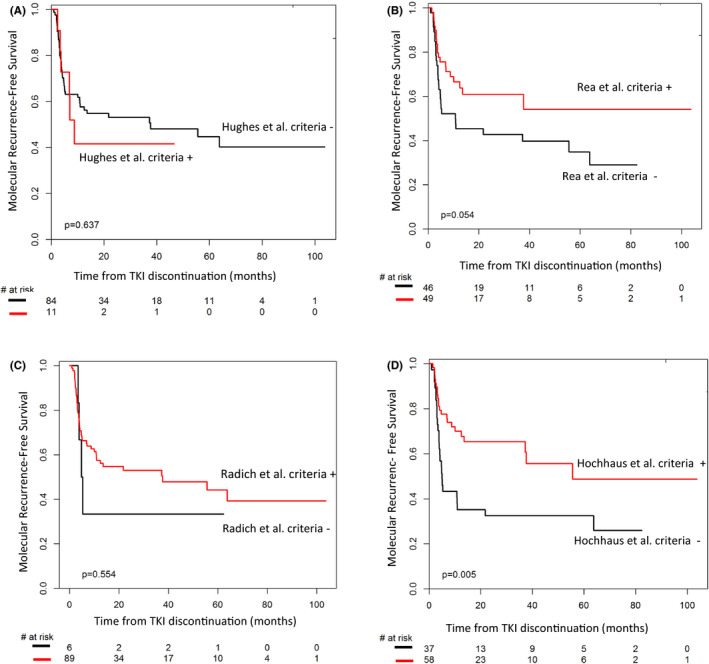
Molecular recurrence‐free survival after TKI cessation according to eligibility criteria proposed in Hughes et al.,^6^ Rea et al.,^7^ Radich et al.,^8^ and Hochhaus et al.,^9^ (*N* = 95 pts)

Among these 95 patients, 11 (11.5%), 49 (51.5%), 89 (93%), 58 (61.0%) fulfilled the selection criteria proposed by Hughes et al.,^6^ Rea et al.,^7^ Radich et al.,^8^ and Hochhaus et al.,^9^ respectively. Baseline and ongoing characteristics of the subgroups defined as optimal for TFR according to selection criteria of recommendations are presented in Table 3. Nine patients were positively selected as optimal candidates according to Hochhaus et al.^9^ criteria but were excluded when Rea et al.^7^ criteria were considered. Among them, seven patients failed to achieve a MMR at 12 months from TKI start (IMA first‐line, *n* = 6; second‐generation (2‐G) TKI first‐line, *n* = 1) and were qualified as Warning according to the 2013 ELN recommendations and two other patients achieved a MR4 sustained at least 36 months without MR4.5 sustained at least 24 months. MRFS of patients who fulfilled the selection criteria proposed by Rea et al.^7^ and Hochhaus et al.^9^ were significantly different as compared to those who did not, whereas no difference of MRFS was observed regarding the criteria proposed by Hughes et al.^6^ and Radich et al.^8^ (Figure 2). The estimated 2‐year MRFS were 41.6% [95% CI: 11.0–72.2] versus 53.1% [95% CI: 42.12–64.07], 61% [95% CI: 46.5–75.5] versus 42.8% [95% CI: 28.3–57.3], 53% [95% CI: 42.2–63.8] versus 33.3% [95% CI: −4.3–70.9], 65.4% [95% CI: 52.7] versus 32.4% [95% CI: 17.3–47.5], for patients who satisfied or not Hughes et al.,^6^ Rea et al.,^7^ Radich et al.,^8^ Hochhaus et al.^9^ eligibility criteria, respectively.

**TABLE 3:** Baseline and ongoing characteristics of the subgroups defined as optimal for treatment free remission according to the selection criteria proposed by Hughes et al.^6^ (*N* = 11 pts), Rea et al.^7^ (*N* = 49 pts), Radich et al.^8^ (*N* = 89 pts), and Hochhaus et al.^9^ (*N* = 58 pts) recommendations among the 95 CP‐CML who discontinued tyrosine kinase inhibitors

Variables	Hughes et al. Blood 2016^6^ *N* = 11	Rea et al. Cancer 2018^7^ *N* = 49	Radich et al. J Natl Compr Canc Netw. 2018^8^ *N* = 89	Hochhaus et al. Leukemia 2020^9^ *N* = 58
Female gender, *n* pts, (%)	5 (45.4)	25 (51.0)	49 (55.1)	30 (51.7)
Median age at diagnosis, years (range)	55.4 (35.9–82.4)	61.6 (27.6–82.4)	61.9 (19.2–85.6)	62.1 (27.6–82.4)
Sokal Risk Score, *n* pts (%)				
Low	4 (36.4)	11 (22.4)	22 (24.7)	13 (22.4)
Intermediate	7 (63.6)	25 (51.0)	44 (49.4)	31 (53.4)
High	0 (0)	10 (20.4)	19 (21.3)	11 (19.0)
Unknown	0 (0)	3 (6.1)	4 (4.5)	3 (5.17)
ELTS Risk Score, *n* pts (%)				
Low	7 (36.4)	26 (53.1)	49 (55.1)	32 (55.2)
Intermediate	4 (63.6)	13 (26.5)	21 (23.6)	14 (24.1)
High	0 (0)	6 (12.2)	13 (14.6)	8 (13.8)
Unknown	0 (0)	4 (8.2)	6 (6.7)	4 (6.9)
BCR‐ABL transcript type, *n* pts (%)				
B2A2	4 (36.4)	15 (30.6)	28 (31.5)	18 (31.0)
B3A2	5 (45.4)	27 (55.1)	52 (58.4)	33 (56.9)
B2A2+B3A2	1 (9.1)	2 (4.1)	3 (3.4)	2 (3.4)
M	1 (9.1)	5 (10.0)	6 (6.7)	5 (8.6)
ACA at diagnosis (major), *n* pts	0 (0)	3 (1)	4 (4.5)	4 (1)
IMA first‐line, *n* pts (%)	10 (90.9)	37 (75.5)	66 (74.2)	45 (77.5)
2‐G TKI first‐line, *n* pts (%)	1 (9.1)	12 (24.4)	23 (25.8)	13 (22.4)
History of WAR, *n* pts (%)	0 (0)	0 (0)	10 (11.2)^a^	7 (12.0)^c^
History of FAIL, *n* pts (%)	0 (0)	0 (0)	4 (4.5)^b^	0 (0)
Achieved MR4.5, *n* pts (%)	11	49	87	58
Median time from TKI start to MR4.5, months (range)	51.2 (8.7–88.0)	23.7 (5.3–88.0)	22.2 (2.6–88.0)	27.1 (5.3–88.0)
Achieved MR4.5 sustained at least 24 months, *n* pts (%)	11 (100.0)	49 (100.0)	82 (92.1)	56 (96.5)
Median MR4.5 duration, months (range)	61.8 (37.1–112.6)	57.0 (24.5–113.4)	44.9 (13.1–113.4)	55.6 (24.5–113.4)
TKI First‐line at TKI discontinuation, *n* pts (%)	8 (72.7)	40 (81.6)	72 (80.9)	47 (82.4)
Median Time from TKI start to TKI discontinuation, months (range)	112.1 (96–134.4)	87.2 (60–134.4)	73.1 (36.0–134.4)	87.7 (59.9–134.4)
Median follow‐up after TKI stop, months (range)	24.8 (5.3–49.7)	24.4 (4.16–103.8)	36.2 (4.16–103.8)	25.8 (4.2–103.8)

Abbreviations: 2‐G TKI, second‐generation TKI; ACA, additional cytogenetic abnormalities; ELTS, EUTOS Long‐Term Survival; FAIL, Failure; IMA, imatinib; MR4, molecular response 4 log; MR4.5, molecular response 4.5 log; TKI, tyrosine kinase inhibitor; WAR, Warning.

^a^ Failed to achieve MMR at 12 months from IMA start, (*n* = 9) or 2‐G TKI start (*n* = 1).

^b^ Failed to achieve or loss of complete cytogenetic response without BCR‐ABL mutation (*n* = 2); BCR‐ABL^IS^ >1% at 12 months from IMA start (*n* = 2).

^c^ Failed to achieve MMR at 12 months from IMA start, (*n* = 6) or 2‐G TKI start (*n* = 1).

Results were more significant (based on *p*‐value) for Hochhaus et al.^9^ than Rea et al.^7^ suggesting a better patients’ categorization by the ELN criteria in our cohort. Same results were observed for MRFR (data not shown).

### 3.5 Molecular remission‐free survival according to selection criteria recommendations and TKI first‐line

**FIGURE 3:**
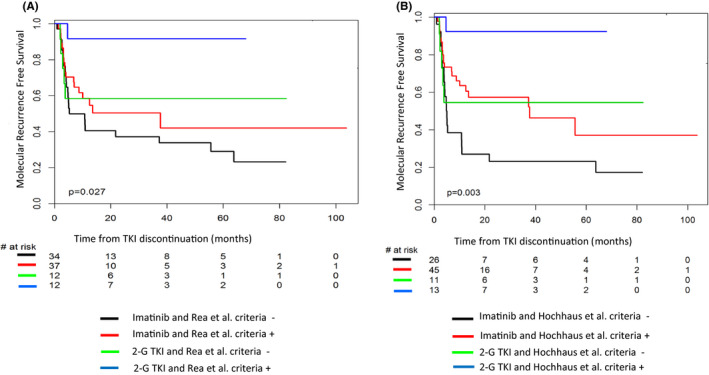
Molecular recurrence‐free survival after TKI cessation according eligibility criteria proposed in Rea et al.,^7^ Hochhaus et al.,^9^ and TKI frontline (IMA vs second‐generation TKI) (*N* = 95 pts)

Based on the hypothesis that first‐line TKI type (IMA vs 2‐3G TKI) may have an impact on MRFS,^13^ we next analyzed the impact of first‐line TKI together with the selection criteria proposed by recommendations. None of the 95 patients who discontinued TKI in this study received frontline or thereafter third generation TKI. As only one patient out of 11 positively selected according to Hughes et al.^6^ criteria and only 1 patient out of 6 negatively selected according to Radich et al.^8^ criteria were treated with 2‐G TKI frontline, analyses were performed using Rea et al.^7^ and Hochhaus et al.^9^ criteria. MRFS according to first‐line TKI type and positive or negative selection with the proposed criteria are presented in Figure 3. Patients treated with 2‐G TKI frontline and who fulfilled the eligibility criteria have the best probability of remaining free from MolRec. In contrast, patients treated with IMA frontline and without eligibility criteria at the time of TKI discontinuation have the highest risk of MolRec.

### 3.6 Second molecular responses after TKI resumption

MolRec occurred in 44/95 (46.32%) patients of whom 38/44 (81.8%) patients had MMR loss. The median time from TKI stop to MolRec was 3.75 months (range: 0.95–63.77) and median maximal *BCR*‐*ABL^IS^* measured during the TFR period in patients who lost MMR was 0.78% (range: 0.101–24.36). None of the MolRec was associated with a loss of complete hematologic response. Except for a 96‐year‐old woman who lost MMR five years after TKI stop but maintained a *BCR*‐*ABL^IS^* <1%, patients who experienced MolRec had TKI resumption.

**FIGURE 4:**
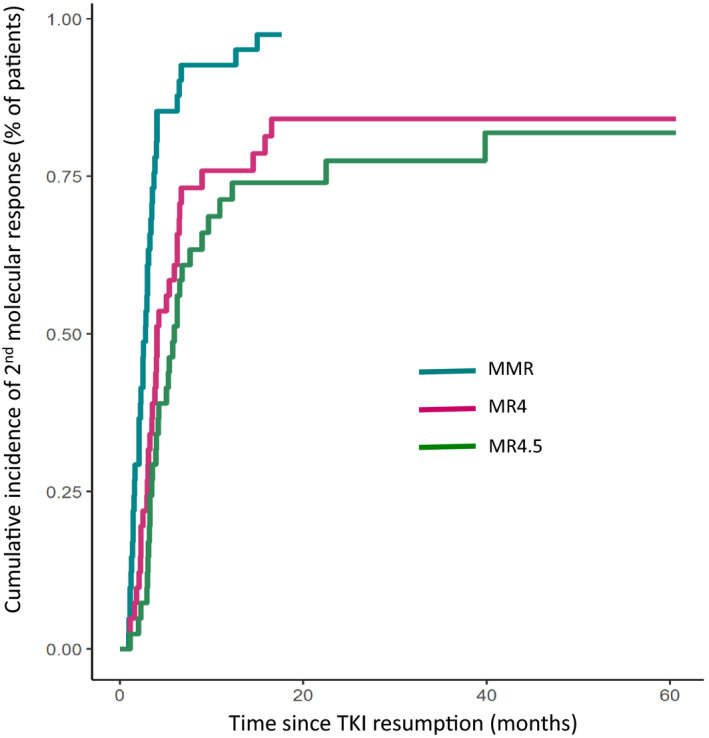
Cumulative incidence of second major molecular response, MR4 log and MR4.5 log after TKI resumption in patients experienced molecular recurrence after TKI stop (*N* = 43 pts)

Thirty‐six (36) (81%) patients resumed the TKI that had been stopped and 7 (19%) patients restarted another TKI. Among them, six patients who have stopped imatinib resumed a 2‐G TKI at the time of MolRec because of chronic grade 2 adverse events experienced on imatinib (mainly chronic arthromyalgia) and one patient who stopped nilotinib resumed dasatinib because of worsening dyslipidemia. The median time from MolRec to TKI resumption and from TKI resumption to last available information were 1.15 months (range: 0.23–6.30) and 37.31 months (range: 2.49–115.87), respectively. Among the 37 patients who restarted TKI after MMR loss, two patients failed to achieve a second MMR because of insufficient follow‐up after TKI resumption (2.49 months) in one patient and admitted lack of adherence following TKI restart in the other one. Six additional patients failed to obtain a second MR4 for several reasons (three patients died of CML unrelated causes at 3.44, 6.49 and 60.39 months after TKI resumption; deaths were related to cardiovascular disease in a 78‐year‐old man who stopped and resumed imatinib; infectious disease in an 86‐year‐old woman who stopped and resumed imatinib; endstage toxic liver disease in a 66‐year‐old woman who stopped imatinib and resumed dasatinib; one patient restarted TKI with a 50% dose‐reduction because of worsening comorbidities; admitted lack of adherence after TKI resumption in two other patients). The CIn of MMR, MR4 and MR4.5 after TKI resumption are presented in Figure 4.

## 4 DISCUSSION

The opportunity of prolonged TKI discontinuation in CP‐CML patients becomes nowadays and in daily clinical practice, a frequently asked question of physicians and patients. Based on the results of numerous clinical trials evaluating the feasibility of TKI stop, recommendations by experts in the field have been suggested and concern notably the optimal selection of the patient, the monitoring after TKI cessation and treatment resumption after MolRec currently defined by MMR loss.^4^, ^6^, ^7^, ^9^ The main objectives of these recommendations were to ensure TKI stop's safety which can be defined by the absence of CML event progression during the TFR period and the achievement of a second DMR after TKI restart following MolRec. Currently, no discontinuation clinical trial reported progression event. A second DMR has been achieved in most if not all the patients after TKI resumption. However, few CML progression cases have been reported after TKI stop outside clinical discontinuation trial.^4^, ^5^ Whether TKI stop accounts for these rare events remains to be determined as sudden blast phases have been previously reported in patients treated with TKI and in apparent sustained complete cytogenetic response and in some of them in DMR.^14^, ^15^, ^16^, ^17^ Nonetheless, these events strongly emphasize the importance of these recommendations. Although 37/95 (39%) patients did not fulfill the selection criteria recently proposed by Hochhaus et al.^9^ no event progression was reported. Nevertheless, we observed several MolRec with a very fast kinetic. Indeed, four and 11 patients at the time of MolRec had a BCR‐ABLIS higher than 10% and 1% respectively. Only 30% of these patients did not fulfill the optimal selection criteria suggested by the recommendations. All these fast MolRec were registered during the six months following TKI cessation and without loss of complete hematologic response at that time, illustrating the need of a close molecular follow‐up after TKI stop. Except for one older patient who did not restart TKI following late MMR loss, all the patients have TKI resumption and only a few of them did not achieve a second DMR (MR4 or MR4.5). Lack of achievement of second DMR was related to patient specific conditions, namely, CML‐unrelated death and or insufficient follow‐up after TKI restart, TKI dose‐reduction because of worsening comorbidities and lack of adherence to TKI after resumption.

Key points of these recommendations concern the optimal selection of the patient before TKI stop. Mainly based on baseline characteristics, TKI duration and duration and depth of DMR observed during TKI treatment, the proposed criteria for an optimal selection of the patient had evolved over time. Indeed, high‐risk Sokal risk score which was associated in some TKI discontinuation studies with a higher risk of MolRec, was considered as an exclusion criterion in the recommendations proposed by Hughes et al. four years ago. However we believe that Sokal risk score and other baseline characteristics such as gender and *BCR*‐*ABL1* transcript type strongly influence the probability of achieving a sustained DMR rather than the risk of relapse after TKI discontinuation. Currently, TKI duration and depth and duration of DMR remain the most important predictive factors of sustained DMR after TKI stop. From a statistical point of view, both variables are correlated and have to be taken into account in a TFR attempt. Whether prolonged TKI duration in patients with sustained DMR, is associated with a higher rate of molecular remission after TKI stop remains to be determined. Interim analyses of the EUROSKI discontinuation trial have suggested that the probability of maintaining MMR after TKI stop increase with years on TKI.^18^ With respect to the size of the subgroup defined by the criteria proposed by Hughes et al.^6^ and based on TKI duration longer than 8 years, molecular remission rate after stop did not increase significantly compared to others despite a sustained DMR in all patients. However, in this subgroup median time from TKI start to the onset of DMR was longer than those observed in the subgroups defined by the criteria proposed by Rea et al.^7^ and Hochhaus et al.^9^ suggesting that time to achieve a DMR may also influence MRFS. Up to date, sustained DMR and TKI duration remain the most relevant predictive factors of MRFS. Although several TKI discontinuation trials had offered the possibility of TKI stop after a minimum TKI duration of 3 years, the median TKI duration before stop ranged from 5 to 8 years with very few patients treated less than 5 years before TKI stop.^1^, ^2^, ^18^, ^19^, ^20^, ^21^ Together these findings highlight that TKI duration before stop has a strong impact on MRFS and reinforces the need for a prolonged TKI duration from the perspective of a successful TKI discontinuation.

Whether patients who did not achieve an optimal TKI frontline response should be excluded from a TFR attempt remains to be determined. In this study, three patients treated with IMA frontline were considered as Failure according to the 2013 ELN recommendations and switched to 2‐G TKI. They achieved a sustained DMR longer than 24 months. Two of them had molecular recurrence and achieved a second DMR after TKI resumption. Seven patients (IMA frontline, *n* = 6) were considered as Warning and did not switch to 2‐G TKI. These patients were excluded from the Rea subgroup but positively selected in the Hochhaus subgroup. Among these "non‐optimal" patients, only two of them experienced molecular relapse after TKI stop. This may explain, in part, the most significant observed *p*‐value when MRFS was compared between patients selected according to Hochhaus et al.^9^ criteria versus Rea et al.^7^ criteria. Thus we believe that patients with a history of warning without switch before stop should not be excluded from TFR attempt provided that DMR has been sustained and TKI duration longer than 5 years.

While aims of TFR recommendations are to help physicians in the selection of the patients and the follow‐up after TKI stop, from the patient's point of view two main questions remain. The first one concerns the probability of meeting the criteria suggested by these recommendations. In this study and according to the most recent recommendations, we can estimate that 36% of the patients will be optimal candidates to TKI discontinuation. The second one concerns the probability of remaining free from TKI after stop if these recommendations are followed. Based on this observational study, we estimate that the probability of MRFS should be close to 60% at 2 years and 50% at 5 years. Meeting the selection criteria suggested by recommendations and front‐line 2‐G TKI leads to the highest MRFS reaching in this study 80%.

## CONFLICT OF INTEREST

GE is a consultant for Novartis, Bristol Myers Squibb, Pfizer, and Incyte Pharma, and has given some lectures for Bristol Myers Squibb, Incyte Pharma, Pfizer, and Novartis. He has received research grants from Novartis and Bristoll Myers Squibb. SD has given some lectures for Novartis and Incyte Pharma. FXM is a consultant for Novartis and Bristol Myers Squibb, and has given some lectures for Bristoll Myers Squibb, Incyte Pharma, Novartis, and Pfizer. He holds a research grant from Novartis. FBa, DA, FL, CD, CL, AS, EK, SM, MPF, FBi, MM, BT, FR, FD, CF, LV, SK, ACN, and AL have nothing to disclose. The authors declare no conflicts of interest for the current work.

## ETHICAL APPROVAL

Patients were identified in the southwest CML register approved by the Comission Nationale Informatique et Libertés (CNIL, Paris, France; CNIL approval DR‐2015–384). All living patients have been informed and have not expressed their opposition to be included in this retrospective and observational study according to the current French regulations. The study has been approved by the Institut Bergonié institutional review board.

## Data Availability

The data that support the findings of this study are available on request from the corresponding author. The data are not publicly available due to privacy or ethical restrictions.
